# The Significance of Motor Evoked Potential Changes and Utility of Multimodality Intraoperative Monitoring in Spinal Surgery: A Retrospective Analysis of Consecutive Cases at a Single Institution

**DOI:** 10.7759/cureus.12065

**Published:** 2020-12-13

**Authors:** Joseph N Frazzetta, Ryan C Hofler, William Adams, Michael J Schneck, G. Alexander Jones

**Affiliations:** 1 Neurosurgery, Loyola University Stritch School of Medicine, Maywood, USA; 2 Neurosurgery, Loyola University Medical Center, Maywood, USA

**Keywords:** intraoperative neuromonitoring, motor evoked potential, positive predictive value, spine surgery

## Abstract

Objective

The objective of the study is to identify specific population groups that may benefit from intraoperative motor evoked potentials (MEP) and to assess positive predictive value (PPV) and negative predictive value (NPV) changes during operation by correlating these with postoperative motor outcomes.

Methods

We retrospectively reviewed 1,043 consecutive patient cases undergoing spine surgery with and without intraoperative monitoring (IOM) at a single institution from January 1, 2016 to December 31, 2017. Demographic and clinical outcome data were collected at multiple time points. An MEP amplitude decrease of 50% or greater was correlated with a motor deficit for this study.

Results

On multivariate analysis, patients with coronary artery disease and who received IOM were more likely to experience no new deficit (p=0.047) than those who did not receive IOM. Additionally, patients with hyperlipidemia and coronary artery disease (CAD) were less likely than those without to experience no new deficit (p=0.001 and p=0.02, respectively). MEP accounted for 244 cases, of which 15 had alert MEP criteria but no deficit for a PPV of 21.05% at day 1 post-operation. Day 7-30 PPV declined to 14.29%, and by day 90, there was no association.

Conclusion

Among patients in our study with CAD, IOM use was associated with significantly better outcomes. Patients with higher intraoperative blood loss, hyperlipidemia, and those with CAD were at increased risk of new neurological deficit. The use of motor evoked potentials was associated with low sensitivity and low PPV.

## Introduction

Neurological complications during spine surgery are rare and include direct spinal cord trauma and cord ischemia. Such events are life-changing and can result in muscle weakness, pain, and even paralysis. The rate of intraoperative complications ranges from 0-3%. However, in spine surgeries of increased risk, such as intradural spinal cord tumors or spine deformity cases, intraoperative complications can be seen at much higher rates [1-4]. Historically, the method to detect intraoperative spinal cord injury was the Stagnara wake-up test, which required anesthetic reversal to observe gross motor function [5]. The subsequent introduction of intraoperative monitoring (IOM) has allowed for earlier detection of irritation and damage of neural elements.

IOM comprises three main categories: motor-evoked potentials (MEP), somatosensory-evoked potentials (SSEP), and electromyography (EMG) [6, 7]. Recent literature has not clearly affirmed the value of IOM during spine surgery, nor the degree to which neurological outcomes are improved as a result of its use [8-11]. Most research on IOM focuses on the sensitivity and specificity of these modalities; however, the positive predictive value (PPV) and negative predictive value (NPV) may better characterize the information provided by IOM. The purpose of this study is to present a single-center, retrospective review of consecutive spine operations, with the goals of identifying specific patient groups that may benefit from multimodality IOM; and specifically assessing the utility of MEP changes during operation as predictors of postoperative motor outcomes via PPV and NPV.

## Materials and methods

Data collection

**Figure 1:**
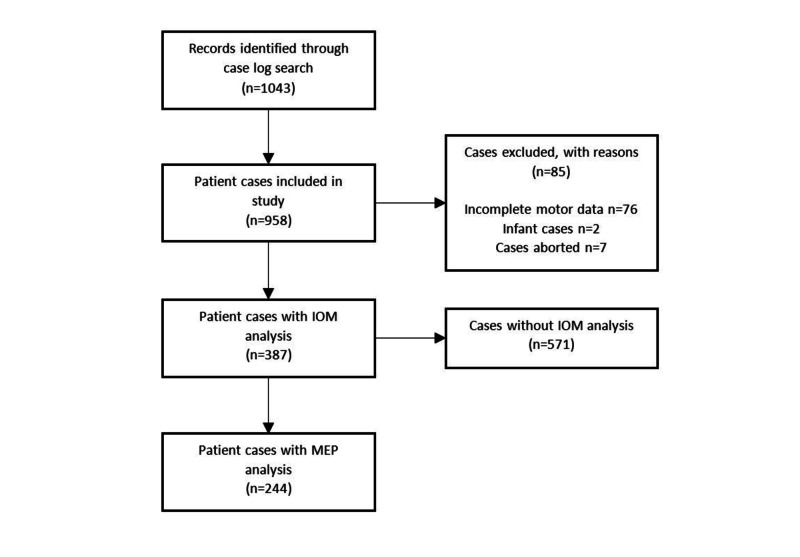
Flowsheet with excluded patients

This study was approved by the Institutional Review Board. Patient consent was not required for this retrospective chart review. All patients undergoing spine surgery at a single institution between January 1, 2016 and December 31, 2017 were included. Patients were identified via the operative case log by one author who did not participate in the care of these individuals. We identified 968 patients undergoing 1,043 procedures and separated them into two groups based on whether or not IOM was used. Demographic data were collected, along with the following variables: site of operation; the number of spine levels operated on; anterior vs. posterior approach; indications for operation; comorbidities; IOM use and which modalities used (SSEP, MEP, and/or EMG); and preoperative and postoperative neurologic outcome data at several time points. Patients with incomplete preoperative data, infant spine cases (under two years of age at time of operation), and patients without data from at least one postoperative visit were excluded from analysis (Figure 1).

Monitoring

A motor evoked potential (MEP) amplitude decrease of 50% or greater is the alert threshold at our institution. By protocol, the operating surgeon is notified of the change, and this is documented in the IOM report. We identified all alerts and correlated these with new onset motor deficit at multiple points postoperatively. We separately studied patients with a sustained MEP change, i.e., one which did not return to at least 50% of the baseline amplitude before the end of the operation. Somatosensory evoked potentials (SSEP) and electromyography (EMG) data were included in our analysis as well.

Statistical analysis

Summary frequencies were displayed as valid counts and proportions for all baseline demographics by intraoperative status. Univariable generalized linear mixed effects models were used to determine the odds of having no new postoperative neurological deficit (defined here as a ‘good neurological outcome’), as a function of treatment characteristics and risk factors. For these models, a binomial distribution with logit link was specified for the motor score, and each model allowed an interaction term to estimate stratified effect sizes at 1, 7-30, and 90 days post-surgery. 

A follow-up multivariable generalized linear mixed effects models was used to estimate the adjusted odds of a good neurological outcome as a function of covariates selected because of their significance on univariable analysis or improvement in model fit statistics. As before, a binomial distribution with logit link was specified for motor score, and this model included an interaction term to allow the association between intraoperative monitoring and the odds of good neurological outcome to depend on patients’ coronary artery disease status. Because patients could contribute multiple procedures to the analysis, random intercepts were allowed for each patient to account for this dependency; a Kenward-Roger correction was used to adjust the denominator degrees of freedom [12].

Sensitivity, specificity, PPV and NPV were estimated with exact confidence limits based on the relationship between an alert threshold change in MEP during operation and the presence of a new post-operative motor deficit. A true positive was defined as a patient with both an MEP alert and new post-operative deficit. A true negative was defined as a patient with no MEP alert and no new post-operative deficit. A false positive was a patient with an MEP alert but no new post-operative deficit, and a false negative was a patient with no MEP alert who experienced a new post-operative deficit. All analyses were completed using SAS version 9.4 (SAS Institute, Cary, USA).

## Results

Patient characteristics, univariable analysis

A total of 968 patients were identified, undergoing 1,043 operations, and attending 3,129 postoperative visits. Among these, 894 patients undergoing 958 procedures had recorded motor examinations prior to their operation, and for at least one day postoperatively. Of these procedures, 387 (40%) underwent some form of IOM (including 244 with MEP), while 571 (60%) underwent an operation without IOM (Table 1). IOM was more likely to be utilized in cases with existing preoperative neurological deficit, cervical or thoracic location, myelopathy, scoliosis or deformity correction, trauma, tumor, and hypertension. IOM was less likely to be used in patients with lumbar location and radiculopathy.

**Table 1:** Baseline demographics by intraoperative monitoring status The average age for the non-intraoperative monitoring group (M=56.22, SD=15.98) was comparable to the average age for the intraoperative monitoring group (M=58.58, SD=17.38; p=0.12). For the whole sample, the average baseline age was 57.18 (SD=16.59). The distribution of blood loss (cc) for the non-intraoperative monitoring group (Mdn=50.00, IQR: 20.00–250.00) was lower than the distribution of blood loss (cc) for the intraoperative monitoring group (Mdn=200.00, IQR: 75.00–500.00; p<0.001). For the whole sample, the median blood loss (cc) was 100.00 (IQR: 25.00–300.00). N  - number of procedures; IQR - interquartile range

	Intraoperative monitoring						p
	No (n=571)		Yes (n=387)		Total (N=958)		
	Count	%	Count	%	Count	%	
Male	305	53.40%	212	54.80%	517	54.00%	0.71
Female	266	46.60%	175	45.20%	441	46.00%	
Pre-operative motor deficit	214	37.50%	191	49.40%	405	42.30%	< 0.001
New post-operative motor deficit	38	6.70%	39	10.10%	77	8.00%	0.06
Cervical location	126	22.10%	202	52.30%	328	34.30%	< 0.001
Thoracic location	77	13.50%	148	38.30%	225	23.50%	< 0.001
Lumbar location	401	70.20%	154	39.90%	555	58.00%	< 0.001
Anterior approach	110	19.30%	82	21.20%	192	20.10%	0.45
Posterior approach	478	83.70%	314	81.30%	792	82.80%	0.56
Myelopathy	124	21.70%	192	49.60%	316	33.00%	< 0.001
Radiculopathy	300	52.50%	144	37.20%	444	46.30%	< 0.001
Deformity / scoliosis	26	4.60%	42	10.90%	68	7.10%	< 0.001
Neurogenic Claudication	93	16.30%	69	17.80%	162	16.90%	0.53
Trauma	51	8.90%	58	15.00%	109	11.40%	0.005
Tumor	8	1.40%	17	4.40%	25	2.60%	0.01
Diabetes	131	22.90%	73	18.90%	204	21.30%	0.19
Hypertension	275	48.20%	214	55.30%	489	51.00%	0.048
Coronary artery disease	62	10.90%	55	14.20%	117	12.20%	0.18
Hyperlipidemia	169	29.60%	133	34.40%	302	31.50%	0.16

**Figure 2:**
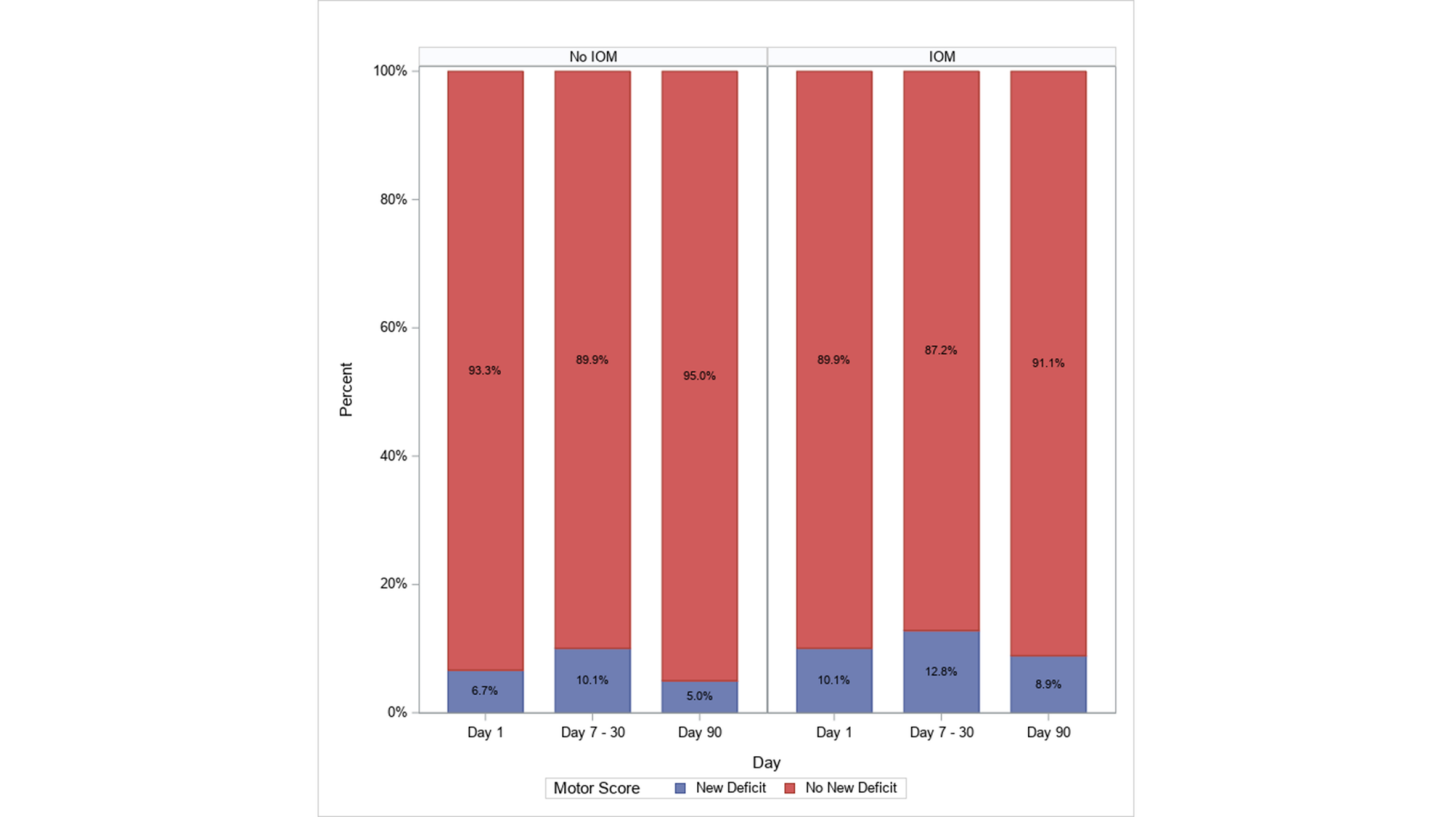
Outcomes breakdown at multiple post-operative points, with and without intraoperative monitoring

On univariable analysis (Table 2 and Figure 2), patients undergoing IOM were less likely to experience a good neurological outcome, though this conclusion was not statistically significant at day 1 (p=0.07), day 7-30 (p=0.28), or day 90 (p=0.07). Conversely, on the first postoperative day, patients with a lumbar location were 1.64 times more likely to have a good neurological outcome than those without a lumbar location (95% CI: 1.01 - 2.66, p=0.047), and patients with neurogenic claudication were 2.53 (95% CI: 1.06 - 6.04) times more likely to have a good outcome than those without neurogenic claudication (p=0.04). Patients with a tumor were less likely than those without a tumor to have a good neurological outcome on the first post-operative day (OR=0.33; 95% CI: 0.11 - 0.99; p=0.048).

**Table 2:** Odds of no new deficit

Days	Odds ratio	95% confidence interval		p
		Lower	Upper	
Intraoperative monitoring: Yes vs. No				
Day 1	0.637	0.393	1.034	0.068
Day 7 – 30	0.769	0.477	1.238	0.279
Day 90	0.544	0.284	1.042	0.066
Pre-operative motor deficit: Yes vs. No				
Day 1	0.872	0.537	1.417	0.581
Day 7 – 30	0.834	0.518	1.342	0.455
Day 90	0.656	0.343	1.255	0.203
Age (per 1-year increase)				
Day 1	1.005	0.991	1.02	0.472
Day 7 – 30	0.986	0.972	1.001	0.063
Day 90	0.981	0.96	1.003	0.082
Sex: male vs. female				
Day 1	0.974	0.6	1.582	0.916
Day 7 – 30	1.003	0.624	1.614	0.989
Day 90	2.003	1.024	3.915	0.042
Cervical location: Yes vs. No				
Day 1	0.633	0.388	1.033	0.067
Day 7 – 30	0.715	0.439	1.163	0.176
Day 90	0.741	0.383	1.435	0.374
Thoracic location: Yes vs. No				
Day 1	0.813	0.47	1.406	0.459
Day 7 – 30	0.775	0.461	1.303	0.336
Day 90	1.083	0.493	2.38	0.843
Lumbar location: Yes vs. No				
Day 1	1.636	1.007	2.656	0.047
Day 7 – 30	1.28	0.796	2.059	0.308
Day 90	1.478	0.773	2.826	0.238
Anterior approach: Yes vs. No				
Day 1	1.133	0.608	2.111	0.694
Day 7 – 30	1.545	0.777	3.073	0.215
Day 90	1.202	0.531	2.718	0.659
Posterior approach: Yes vs. No				
Day 1	0.979	0.515	1.863	0.949
Day 7 – 30	0.591	0.28	1.246	0.167
Day 90	0.982	0.432	2.233	0.966
Myelopathy: Yes vs. No				
Day 1	0.636	0.389	1.041	0.072
Day 7 – 30	0.567	0.349	0.923	0.022
Day 90	0.816	0.419	1.59	0.549
Radiculopathy: Yes vs. No				
Day 1	1.096	0.674	1.781	0.713
Day 7 – 30	0.981	0.611	1.577	0.938
Day 90	0.55	0.285	1.059	0.074
Deformity / scoliosis: Yes vs No				
Day 1	1.998	0.596	6.696	0.262
Day 7 – 30	0.822	0.361	1.87	0.639
Day 90	1.18	0.34	4.095	0.794
Neurogenic claudication: Yes vs. No				
Day 1	2.534	1.063	6.037	0.036
Day 7 – 30	1.614	0.813	3.204	0.172
Day 90	0.818	0.371	1.804	0.618
Trauma: Yes vs. No				
Day 1	0.945	0.446	2.003	0.882
Day 7 – 30	0.998	0.48	2.073	0.996
Day 90	4.897	0.651	36.826	0.123
Tumor: Yes vs. No				
Day 1	0.333	0.112	0.989	0.048
Day 7 – 30	1.361	0.294	6.302	0.693
Day 90	0.25	0.046	1.369	0.11
Diabetes: Yes vs. No				
Day 1	0.806	0.458	1.418	0.453
Day 7 – 30	0.669	0.391	1.145	0.142
Day 90	1.204	0.531	2.729	0.657
Hypertension: Yes vs. No				
Day 1	1.076	0.664	1.744	0.767
Day 7 – 30	0.717	0.444	1.157	0.173
Day 90	1.112	0.583	2.122	0.746
Coronary artery disease: Yes vs. No				
Day 1	0.657	0.34	1.272	0.213
Day 7 – 30	0.87	0.448	1.692	0.682
Day 90	0.42	0.185	0.957	0.039
Hyperlipidemia: Yes vs No				
Day 1	0.714	0.433	1.177	0.186
Day 7 – 30	0.767	0.47	1.251	0.288
Day 90	0.332	0.172	0.641	0.001
Monitorable: Yes vs No				
Day 1	0.631	0.389	1.024	0.062
Day 7 – 30	0.802	0.497	1.292	0.364
Day 90	0.539	0.282	1.033	0.062
Estimated blood loss (per 100cc increase)				
Day 1	1.043	0.97	1.122	0.255
Day 7 – 30	0.944	0.901	0.99	0.017
Day 90	0.987	0.917	1.063	0.735

On postoperative days 7-30, each additional 100cc intraoperative blood loss correlated with a 5.6% decrease in the odds of a good neurological outcome (OR=0.94, 95% CI: 0.90 - 0.99; p=0.02), and patients with myelopathy were only 0.57 (95% CI: 0.35 - 0.92) times as likely to experience a good outcome as those without myelopathy (p=0.02).

On postoperative day 90, males were more likely than females to experience a good outcome (OR=2.00, 95% CI: 1.02 - 3.91; p=0.04). Patients with coronary artery disease were less likely to have a good outcome (OR=0.42, 95% CI: 0.19 - 0.96; p=0.04), as were patients with hyperlipidemia (OR=0.33, 95% CI: 0.17 - 0.64; p=0.001).

Patient characteristics, multivariable analysis

On multivariable analysis, patients with coronary artery disease who received IOM were 2.59 (95% CI: 1.01 - 6.65) times more likely than those who did not receive IOM to experience a good neurological outcome (p=0.047, Table 3). Patients with hyperlipidemia were significantly less likely than those without hyperlipidemia to experience a good outcome (OR=0.65, 95% CI: 0.45 - 0.93; p=0.02).

**Table 3:** Adjusted odds of no new deficit Valid N = 966. The multivariable estimates are also adjusted for physician type (ortho vs. neuro). IOM - intraoperative monitoring (IOM); CAD - coronary artery disease

	Adjusted odds ratio	95% confidence interval		p
		Lower	Upper	
IOM*CAD interaction				0.02
IOM: Yes versus No | CAD = Yes	2.594	1.012	6.645	0.047
IOM: Yes versus No | CAD = No	0.883	0.529	1.475	0.63
CAD: Yes versus No | IOM = Yes	1.450	0.724	2.904	0.29
CAD: Yes versus No | IOM = No	0.494	0.269	0.906	0.02
Day				0.02
Day 7-30 versus Day 1	0.721	0.517	1.006	0.054
Day 90 versus Day 1	1.218	0.817	1.817	0.33
Day 90 versus Day 7-30	1.689	1.134	2.516	0.01
Claudication: Yes versus No	2.242	1.379	3.644	0.001
Hyperlipidemia: Yes versus No	0.646	0.451	0.926	0.02

The odds of a good outcome were significantly higher by postoperative day 90 than postoperative day 7-30 (OR=1.69, 95% CI: 1.13 - 2.52; p=0.01); that is, patients with a deficit at postoperative day 7-30 saw this deficit resolve by postoperative day 90. Those with claudication were significantly more likely than those without claudication to experience a good outcome (OR=2.24, 95% CI: 1.38 - 3.64; p=0.001).

Intraoperative MEP changes

A total of 244 cases with at least one day of follow-up were available to assess the sensitivity, specificity, negative predictive value, and positive predictive value of an MEP alert change to detect a new deficit (Table 4). Among these patients, the prevalence of a new deficit on the first post-operative day was 12.7% (n=31), and only four (1.6%) were true positive cases leading to imprecise estimates of sensitivity (12.90%; 95% CI: 3.63 - 29.83) and PPV (21.05%; 95% CI: 6.05% - 45.57%). Conversely, 198 (81%) patients were true negative cases leading to estimates of specificity (92.96%; 95% CI: 88.65% - 96.01%) and NPV (88.00%; 95% CI: 83.02% - 91.94%).

**Table 4:** Epidemiology scores for motor evoked potential change and post-operative motor scores Valid N - the number of patients used to calculate the estimates. These epidemiology scores are restricted to patients’ first surgery.

	Valid N	Estimate	Exact 95% confidence interval	
			Lower	Upper
Day 1	244			
Sensitivity		12.90	3.63	29.83
Specificity		92.96	88.65	96.01
Positive predictive value		21.05	6.05	45.57
Negative predictive value		88.00	83.02	91.94
False positive probability		78.95	54.43	93.95
False negative probability		12.00	8.06	16.98
Day 7-30	203			
Sensitivity		7.14	0.88	23.50
Specificity		93.14	88.33	96.41
Positive predictive value		14.29	1.78	42.81
Negative predictive value		86.24	80.50	90.81
False positive probability		85.71	57.19	98.22
False negative probability		13.76	9.19	19.50
Day 90	162			
Sensitivity		0.00	--	--
Specificity		93.10	87.68	96.64
Positive predictive value		0.00	--	--
Negative predictive value		88.82	82.70	93.35
False positive probability		100.00	69.15	100.00
False negative probability		11.18	6.65	17.30

By day 7-30, 203 patients had follow-up data. The prevalence of a new deficit was 13.8% (n=28), and only two (1.0%) were identified as true positive cases yielding imprecise estimates of sensitivity (7.14%; 95% CI: 0.88% - 23.50%) and PPV (14.29%; 95% CI: 1.78% - 42.81%). As before, a large number of patients (n=163 or 80%) were true negative cases, yielding estimates of specificity (93.14%; 95% CI: 88.33% - 96.41%) and NPV (86.24%; 95% CI: 80.50% - 90.81%).

Finally, 162 patients had follow-up data available by day 90. For these patients, the prevalence of a new deficit was 10% (n=17), and there were no true positive cases. However, 135 (83%) patients were true negative cases leading to estimates of specificity (93.10; 95% CI: 87.68 - 96.64) and NPV (88.82%; 95% CI: 82.70 - 93.35).

Of all patients with MEP change during operation, only three had an amplitude drop that persisted until the end of the operation. Among these three patients, none had a postoperative motor deficit, rendering the sensitivity and PPV zero for a sustained MEP dropout during the operation (Table 5).

**Table 5:** Epidemiology scores for persistent motor evoked potential change and post-operative motor scores Valid N - the number of patients used to calculate the estimates. These epidemiology scores are restricted to patients’ first surgery.

	Valid N	Estimate	Exact 95% confidence interval	
			Lower	Upper
Day 1	228			
Sensitivity		0.00	--	--
Specificity		98.51	95.70	99.69
Positive predictive value		0.00	--	--
Negative predictive value		88.00	83.02	91.94
False positive probability		100.00	29.24	100.00
False negative probability		12.00	8.06	16.98
Day 7-30	191			
Sensitivity		0.00	--	--
Specificity		98.79	95.69	99.85
Positive predictive value		0.00	--	--
Negative predictive value		86.24	80.50	90.81
False positive probability		100.00	15.81	100.00
False negative probability		13.76	9.19	19.50
Day 90	153			
Sensitivity		0.00	--	--
Specificity		99.26	95.97	99.98
Positive predictive value		0.00	--	--
Negative predictive value		88.82	82.70	93.35
False positive probability		100.00	2.50	100.00
False negative probability		11.18	6.65	17.30

## Discussion

Patient characteristics and use of IOM

The use of monitoring at our institution is at the discretion of the operating surgeon. In general, it is more likely to be used in cases with a higher risk of neurological injury, including myelopathy, tumor, and deformity, and less likely in cases of isolated lumbar stenosis. Thus, our finding that patients undergoing IOM were more likely to have a new neurological deficit than those without IOM, while not statistically significant, must be interpreted considering this selection bias: surgeons preferentially monitor the cases at higher risk of deficit. This also accounts for better outcomes in patients with lumbar stenosis and neurogenic claudication.

Our results suggest that coronary artery disease is not only associated with increased risk of postoperative deficit but that patients in this group with IOM fared better than those without. Additionally, hyperlipidemia and increasing blood loss were risk factors for the new neurological deficit. Given the methodology of our study and the lack of physiologic rationale to support them, we think it is best to assume these findings are spurious.

It is no surprise that neurologic deficits were more common at day 1 and days 7-30 following operation than at day 90. A number of neurologic deficits, including C5 palsy, will improve with time.

Intraoperative MEP changes

Ideally, IOM will allow a surgeon to perform a safer operation by alerting the surgeon of impending or early neurological compromise before it leads to a permanent deficit. The best hope for this, in our opinion, is the use of motor evoked potentials (MEP), which provide real-time feedback on the function of the corticospinal tracts at the level of the spinal cord.

For this modality, our data were very disappointing. On postoperative day 1, we saw new deficits in 31 out of 244 patients. However, only four of these had MEP changes during the operation, yielding a sensitivity of 12.9%. For all 19 patients with an MEP change during the operation, the PPV was just 21.05%. The PPV dropped to 14.29% at day 7-30, and zero by day 90. While we expected the PPV to be low and the NPV high (indicating a high number of false positive tests), we were surprised that the sensitivity was so low, especially considering that our alert threshold is an amplitude drop of just 50%, while many others use 80%, 90%, or even 100% [13, 14].

The statistics we choose to analyze are really at the core of this analysis. Many other series report on the sensitivity and specificity of MEP changes during operation. However, for two reasons, these numbers are less relevant to the operating surgeon than PPV and NPV.

First, sensitivity and specificity are easier concepts to grasp but generally less useful than PPV and NPV. Still, sensitivity and specificity have some role in helping a clinician to decide what diagnostic test to order before ordering the test when testing for a disease with high prevalence. The ideal screening test for a given disease, for example, will have high sensitivity and low cost. It can be widely deployed, will accurately identify everyone who has (or might have) a disease, and do so at a low cost. For such a screening test, specificity is much less important. However, a confirmatory test with high specificity is necessary to exclude the false positives generated by the screening test.

A change in MEP is a different matter than a screening test. In this case, the surgeon is presented with the result of a test and needs to know how to interpret this; PPV and NPV are better suited to help the surgeon understand the given result than sensitivity and specificity. The ideal test would have 100% PPV, meaning all positive results are true positives. Applied to IOM, this means any MEP drop during the case would indicate a true neurological insult and would not be caused by anesthetic factors, room temperature, positional ischemia or neuropraxia, or any other spurious input. It would also have 100% NPV, with all negative results being true negatives. This means that any patient without MEP change during the operation would emerge from the operation with no new deficit. Unfortunately, a low PPV, as we found in this series, means that most MEP alerts are false alarms. Stating the initial PPV of 21.05% another way, nearly four out of five patients with an intraoperative drop in MEP awoke with no new deficit whatsoever. In each case, a great deal of time (and no small amount of stress for the surgeon and OR team) is given over to treat a 'problem' that really doesn’t exist.

Second, PPV and NPV are dependent on the prevalence of a condition, whereas sensitivity and specificity are not. The lower the prevalence of the condition (in this case, a new neurological deficit), the lower the PPV. In this series, the risk of new neurologic deficit drops to 10.5% by 90 days (including a number of new C5 palsies); with such a low prevalence, the PPV will be low as well. Understanding the interdependence of PPV and prevalence is very important when interpreting results (such as an MEP drop) in a test designed to screen for something of very low prevalence (new neurologic deficit after the operation).

This might argue for using MEPs only during the cases with the highest risk (i.e., the highest prevalence) of postoperative neurologic deficit, which should lead to a higher PPV and thus greater utility for the surgeon. However, the existing literature has not shown a clear benefit to monitoring in cases such as spinal cord tumor resection [15-17].

Our study, and the larger body of literature on this topic, clearly highlight the need for a prospective trial to determine the utility of IOM. However, several challenges must be overcome.

For any topic to warrant the effort and expense of a prospective trial, whether randomized or single-arm, there must exist clinical equipoise regarding the treatment. IOM carries additional burdens in terms of cost, and duration of the operation, and of anesthesia. The payoff for this, at least in theory, is to allow the surgeon a chance to prevent an iatrogenic neurological injury or correct a temporary insult before it becomes a permanent injury. This benefit remains elusive, at least in the literature published to date. We think equipoise really does exist in the field of IOM.

Further challenges for a prospective trial exist. The blinding of the surgeon to the results of data being collected in real time raises serious ethical questions. Additionally, logistical issues involving the monitoring technician and interpreting physician being sent away on the morning of an operation when the patient is randomized to the control arm could be problematic.

The best solution might be a single-arm trial, looking only at patients undergoing IOM. The focal point of this would be identifying cases in which a loss of MEP signals can be reversed by the surgeon and/or the anesthesiologist, ideally following a standardized, and predetermined, protocol. This data could be used to calculate the cost of IOM on the basis of each injury avoided and the number needed to treat (NNT) to prevent one injury. However, the validity of such a study depends on at least the high sensitivity of IOM, which remains an open question.

Limitations

The primary limitation of this study is its retrospective design. IOM use was based on the attending surgeon’s discretion, leading to potential selection bias and heterogeneity in terms of which cases they chose to monitor or not. Also, we do not have a mechanism to identify which cases had an intraoperative MEP drop that was successfully addressed and rectified by the surgeon. The sensitivity was low enough that this was not a critical matter in data analysis. However, distinguishing between MEP alerts due to neural element compromise and those due to anesthetic or other factors would be important in any prospective trial.

## Conclusions

Our study demonstrates the significance of an intraoperative MEP change when considered in the context of the low prevalence of true neural element injury. The use of motor evoked potentials (MEP) to predict or prevent neurological injury was associated with low sensitivity and low PPV on postoperative day 1; by postoperative day 90, there was no correlation between MEP change and motor outcome. Additionally, we have identified specific patient groups who may benefit from IOM. Patients with coronary artery disease had a higher risk of a new postoperative neurological deficit than those without. Among patients with CAD, the use of IOM was associated with significantly better outcomes. Patients with higher intraoperative blood loss and those with hyperlipidemia were at increased risk of new neurological deficit. This paper adds to our understanding of the strengths and limitations of IOM and further illustrates the need for a prospective trial to clarify the value of IOM in spine surgery.
